# Effect of microgravity on the solidification of aluminum–bismuth–tin immiscible alloys

**DOI:** 10.1038/s41526-019-0086-z

**Published:** 2019-11-18

**Authors:** Hongxiang Jiang, Shixin Li, Lili Zhang, Jie He, Jiuzhou Zhao

**Affiliations:** 1 Institute of Metal Research, Chinese Academy of Sciences, Shenyang, 110016 China; 2 School of Materials Science and Engineering, University of Science and Technology of China, Shenyang, 110016 PR China

**Keywords:** Materials science, Physics

## Abstract

Directional solidification experiment was carried out with Al-Bi-Sn immiscible alloy under microgravity environment onboard the Tiangong 2 space laboratory of China. Sample with a well-dispersed microstructure was obtained by properly designing the experimental scheme, the matrix shows equiaxed morphology, and there is no visible gas cavity or pinhole in the sample. In contrast, the reference samples solidified on earth show phase-segregated structure and contain some gas cavities or pinholes. The grain morphology of the terrestrial sample depends on the solidification direction, it is equiaxed when the sample ampoule was withdrawn against the gravity direction, while it is columnar when the sample ampoule was withdrawn along the gravity direction. The solidification process and affecting mechanisms of microgravity on the microstructure formation are discussed. The results indicate that the microgravity conditions can effectively diminish the convective flow of the melt and the Stokes motions of the minority phase droplets and gas bubbles, which are helpful for suppressing the occurrence of macro-segregation and preventing the formation of porosity. The results also demonstrate that the microgravity conditions favor the detachment between the melt and the wall of crucible, thus increasing the nucleation undercooling of α-Al nuclei and promoting the formation of equiaxed grain.

## Introduction

Immiscible alloys show a phase diagram characterized by the appearance of a miscibility gap in the liquid state.^1–8^ They are especially suitable for the manufacturing of either in situ particle composite materials or composite materials with a core/shell structure and thus have a strong industry application background.^9–13^ For instance, Al-Bi alloy can be used as the advanced bearings if the soft Bi phase disperses uniformly in a comparatively hard Al-based matrix,^14,15^ and Cu-Fe and Cu-Cr alloys are high-strength, high-electrical-conductivity materials.^16–19^ However, these alloys have an essential drawback, in that the miscibility gap poses problems during solidification. When a single-phase melt of immiscible alloy is cooled into the miscibility gap, it decomposes into two liquids enriched with different components. Generally, the liquid–liquid decomposition begins with the nucleation of the minority phase (MP). These nucleated droplets then grow/coarsen by solute diffusion, settle or float due to specific gravity differences between phases, and migrate due to the temperature gradient and the convective flow of melt. The droplet motions lead to the formation of a phase-segregated microstructure when these alloys are solidified on earth. The applications of immiscible alloys are thus seriously limited.^20–24^

Microgravity is an excellent environment to inhibit the convection.^25–28^ Since the 1970s, solidification experiments have been carried out under microgravity conditions of space to obtain immiscible alloys with a dispersed microstructure. For example, in 1978, Carlberg et al.^29^ performed solidification experiments with the Zn-Bi immiscible alloys on the second TEXUS flights and found that the phase macro-segregation occurs during the solidification. This indicates that the gravity intensity is not the unique factor determining the phase-segregation process. Potard^30^ investigated the solidification process of Al-In alloys under microgravity during the NASA-SPAR IX flight of 20 January 1981, and the results indicated that the sample showed an In-rich-core/Al-rich-shell structure. The author attributed the formation of such a structure to the wetting behavior of the liquid phases to the crucible. Gells et al.^31^ also carried out the solidification experiments with Al-In alloys during the SPAR II rocket flight, but the samples still showed a phase-segregated structure. They concluded that the phase segregation was induced by the Marangoni convection in the melt. Huang et al.^32^ and Fujii et al.^33^ investigated the effect of microgravity on the solidification of Al-Bi and Al-Pb-Bi alloys, respectively. Their results demonstrated that the macro-segregation caused by the density difference between the MP droplets and the matrix liquid phase was diminished. However, the distribution of the MP in the whole sample was still not homogeneous and there were large voids in the samples or deep, irregular helical grooves on the surface of the sample. Since then, great progresses have been made in this field. Nevertheless, the sample solidified under microgravity conditions in space usually shows a segregated microstructure and contains grooves or gas cavity defects, and the microstructure evolution during cooling an immiscible alloy under the microgravity condition remains a scientific problem to be further probed.

In this paper, one directional solidification experiment was carried out with Al-Bi-Sn immiscible alloy under microgravity environment onboard Chinese Tiangong 2 space laboratory on October 24–25, 2016. The hold temperature and withdrawn velocity are ~950 K and 28 μm/s, respectively. For the detailed procedure, please refer to the “Methods” section at the end of the paper. Two referenced experiments were performed by following the same procedure except for the withdrawn direction of sample ampoule. For one of the terrestrial experiments, the ampoule was pulled out from the experimental furnace along the gravity direction during the solidification process (called “gravitational solidification”). For the other one, the ampoule was withdrawn against the gravity direction (called “anti-gravitational solidification”). The influences of the microgravity on the solidification process and structures of immiscible alloy are discussed in detail.

## Results

**Fig. 1:**
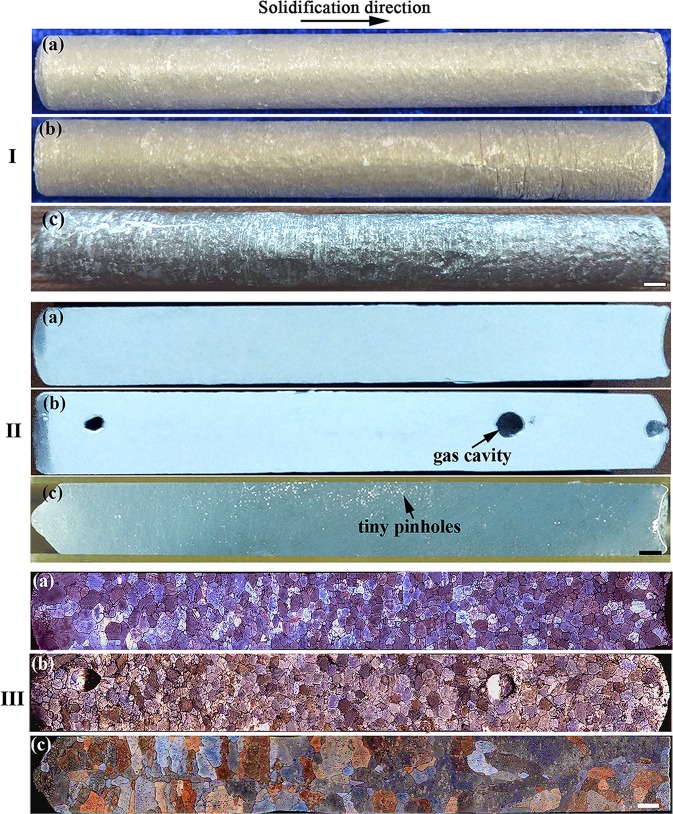
As-cast surface appearance **I**, longitudinal sections **II**, and matrix grain morphologies **III** of the Al-Bi-Sn samples solidified in space **a**, anti-gravitationally on earth **b**, and gravitationally on earth **c**. Scale bar = 2 mm

Figure 1I shows the surface appearances of the samples. It can be seen that the surface of the space sample is smooth, while the surfaces of the two terrestrial samples are rough and display many grooves. Table 1 shows the diameters at different positions along the longitudinal direction of the samples. It demonstrates that the diameter of the sample solidified in space is almost uniform, while there are differences in the diameters at different positions of the terrestrial samples.

**Table 1:** Diameters of different positions, densities, and the average grain size of the Al-Bi-Sn samples solidified in space and on earth

Sample	Space sample	Anti-gravitational solidification sample	Gravitational solidification sample
Average diameters and standard deviation of different positions^a^ (mm)	6.85, 0.016	6.88, 0.025	7.10, 0.004
	6.84, 0.012	6.89, 0.016	6.99, 0.022
	6.85, 0.014	6.95, 0.023	6.93, 0.018
	6.83, 0.015	6.97, 0.025	6.88, 0.032
	6.83, 0.019	7.04, 0.029	6.78, 0.016
Density^b^ (g/cm^3^)	2.726	2.710	2.721
Measurement uncertainty of the density	0.0022	0.0029	0.0042
Average grain size (μm)	413.6	447.2	739.4

^a^Five equivalent distance points from the initial solidified end to the final solidified end of the samples were chosen, the distance between two adjacent points is ~14 mm. Each value of the diameters is the average value of 4 measured results

^b^The densities of the sample were measured by using the Archimedes’ Drainage Method. Each value of the densities is the average value of 4 measured results

Figure 1II shows the longitudinal sections of the Al-Bi-Sn samples. There is no visible gas cavity in the sample solidified in space. In contrast, there are several large gas cavities in the anti-gravitational solidification sample and some tiny pinholes exist in the gravitational solidification sample; in order to check the reproducibility of such a phenomena, two additional experiments were carried out on earth by following the same procedure, and similar results were obtained. Quantitative metallographic analysis indicates that the average sizes of the gas cavities in the anti-gravitational solidification sample and the tiny pinholes in the gravitational solidification sample are about 1.64 and 0.11 mm, respectively. The result is consistent with the fact that the density of the sample solidified in space is larger than the densities of the terrestrial samples (see Table 1).

Figure 1III shows the grain morphologies of the Al-Bi-Sn samples solidified in space and on earth. It is indicated that almost all the α-Al grains show equiaxed grain morphology for the sample solidified in space and the anti-gravitational solidification sample, but the columnar grains (the aspect ratio >2) account for about 32% in the gravitational solidification sample (the columnar grains aligned to the axis account for about 16.2% and the columnar grains pointing toward the axis account for about 15.8%). Analysis results demonstrate that the average grain size of the space sample is about 413.6 μm, which is ~7.5% less than that of the anti-gravitational solidification sample and ~44% less than that of the gravitational solidification sample (see Table 1).

**Fig. 2:**
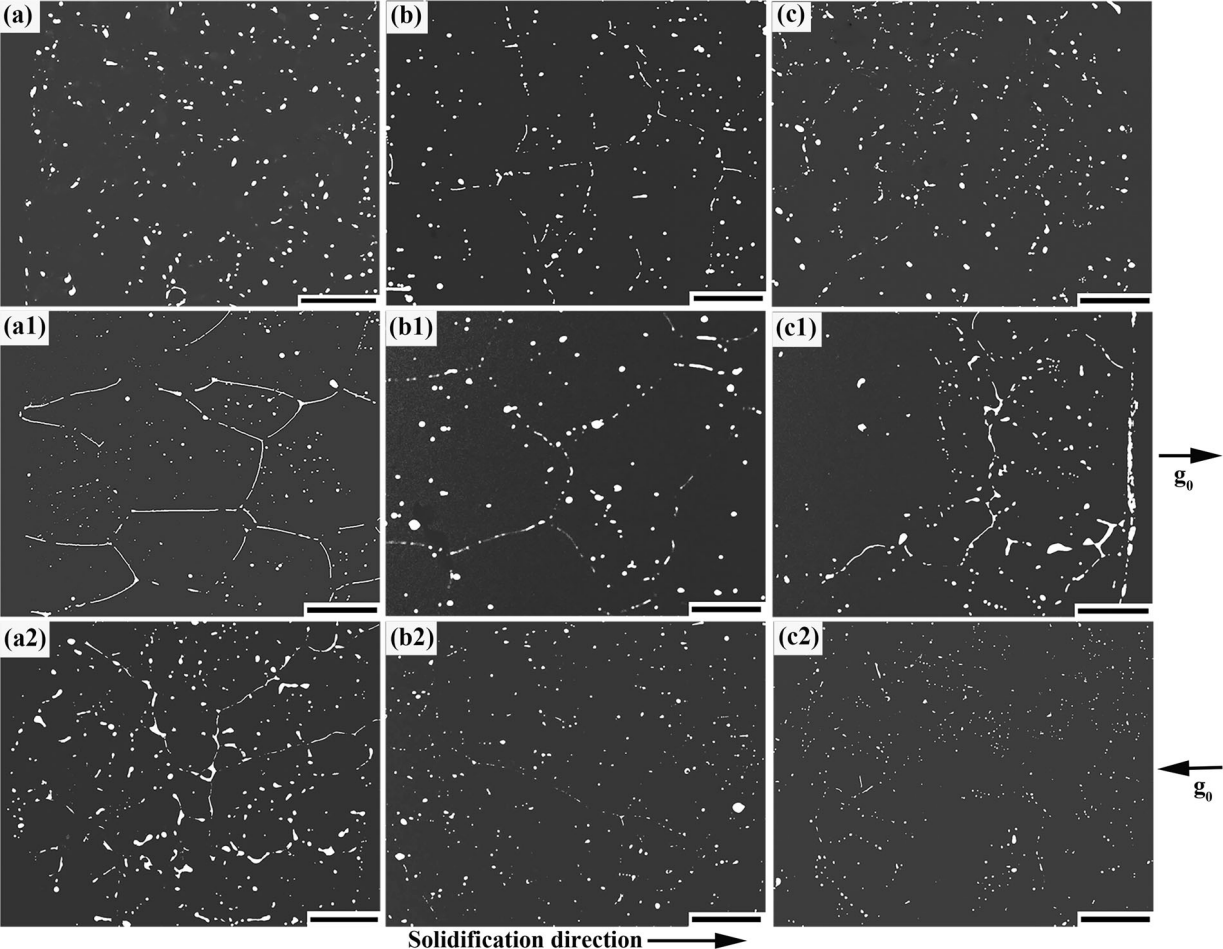
SEM images of the microstructure along the axial direction of the space sample (**a**–**c**), anti-gravitational solidification sample (**a1**–**c1**), and gravitational solidification sample (**a2**–**c2**). **a**, **a1**, **a2** are the initial solidification parts of the samples. **b**, **b1**, **b2** are the middle parts of the samples. **c**, **c1**, **c2** are the final solidification parts of the samples. Dark and bright areas correspond to the aluminum matrix and (Bi,Sn)-rich particles, respectively. Scale bar = 400 μm

**Fig. 3:**
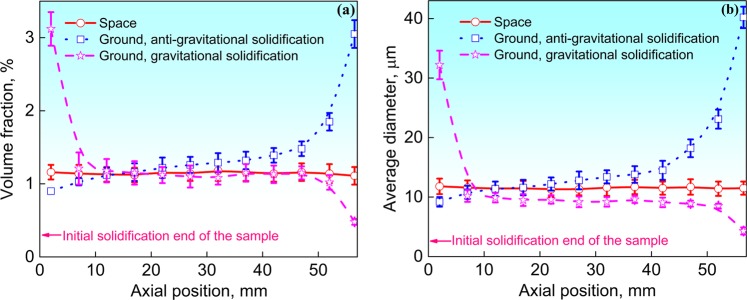
Volume fraction **a** and average diameter **b** of the (Bi,Sn)-rich phase along the axial direction of the Al-Bi-Sn samples. Error bars represent standard deviation

Figure 2 shows the scanning electron microscopic (SEM) images of the microstructures along the axial positions of samples. Energy dispersive X-ray spectrometry (EDS) analyses revealed that the white MP is (Bi,Sn)-rich phase and the matrix is α-Al. Figure 3 shows the volume fraction and average diameter of the (Bi,Sn)-rich phase along the axial (*z*) direction of the samples. These results demonstrate that the sample solidified in space exhibits well-dispersed distribution of (Bi,Sn)-rich phase throughout the whole sample. In contrast, the spatial distributions of the (Bi,Sn)-rich phase in the two samples solidified on earth are both remarkably non-uniform.

**Fig. 4:**
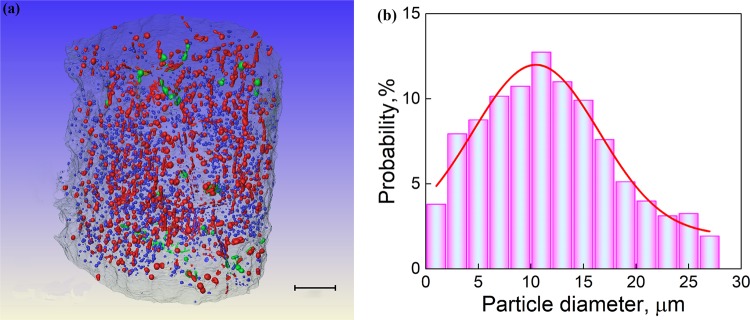
High-resolution XRT image at the middle of the Al-Bi-Sn sample solidified in space **a** and the typical size distribution of (Bi,Sn)-rich phase particles **b**. Scale bar = 200 μm

Figure 4 shows the high-resolution X-ray tomography (XRT) image at the middle of Al-Bi-Sn sample solidified in space and the typical size distribution of the MP. The resolution of the XRT system is 1 μm. The results indicate that the size of the MP ranges from about 1.5 to 28 μm.

## Discussions

For a given part of the Al-Bi-Sn immiscible alloy sample, its liquid–liquid phase transformation process is as follows. The initial homogeneous single-phase melt becomes supersaturated when the temperature is below the binodal line of the alloy. Then the supersaturation increases continuously with the drop of the temperature until a critical value is attained such that the nucleation of the MP begins. After nucleation, these droplets grow, coarsen, and migrate in the melt. The Stokes motion and Marangoni migration velocities of a MP can be calculated by^34^:

1 $${\mathbf{u}}_{\rm{S}} = \frac{{2{\it{g}}(\rho _{\it{\upbeta }} - \rho _{\rm{m}})}}{3}\frac{{\eta _{\rm{m}} + \eta _{\it{\upbeta }}}}{{\eta _{\rm{m}}(2\eta _{\rm{m}} + 3\eta _{\it{\upbeta }})}}R^2$$

2 $${\mathbf{u}}_{\rm{M}} = - \frac{{2R}}{{[(2{\it{k}}_{\rm{m}} + {\it{k}}_\beta )/{\it{k}}_{\rm{m}}](2\eta _{\rm{m}} + 3\eta _\beta )}}\frac{{\partial \sigma }}{{\partial {\it{T}}}}\nabla {\it{T}}$$

where *T* is the temperature, *R* is the radius of the MP, *ρ*_m_ and *ρ*_*β*_ are the densities of the matrix and the MP, respectively, *η*_m_ and *η*_*β*_ are the viscosities of the matrix and the MP, respectively, *k*_m_ and *k*_*β*_ are the thermal conductivities of the matrix liquid and the MP, *σ* is the interfacial energy between the matrix melt and the MP, and ∇*T* is the temperature gradient. The values of these parameters can be found in refs. ^35,36^

Under the directional solidification conditions, an immiscible alloy forms a well-dispersed microstructure only if a steady state of solidification is established. In order to establish the steady state of solidification, the MP of all sizes and located at any position in front of the solidification interface must move toward the solidification interface^36–38^ or the motion velocity of the MP relative to the solidification interface (***u***^*z*^) satisfies:

3 $${\mathbf{u}}_{\forall (R,r,z)}^z = {\mathbf{u}}_{\rm{M}}^z + {\mathbf{u}}_{\rm{S}} + {\boldsymbol{V}}_0 + {\boldsymbol{V}}_{\rm{C}}^z < 0$$

where ***V***_0_ is the withdrawing velocity of the ampoule whose direction is defined as the negative direction and ${\mathbf{u}}_{\rm{M}}^z$ is the *z*-component of the Marangoni migration velocity of the droplet. ${\boldsymbol{V}}_{\rm{C}}^z$ is the Buoyancy-driven convective flow in the melt along the axial direction of the sample. The strength of the convection flow can be estimated by the Rayleigh number (Ra), which is given by^39^:

4 $${\rm{Ra}} = \frac{{g\beta \Delta TL^3}}{{\nu ^2}} \cdot \frac{\nu }{\alpha }$$

where *β* is the coefficient of volume expansion, Δ*T* is the temperature difference between the upper and lower interfaces of the melt, *L* is the depth of the melt, *α* is the thermal diffusivity, and *ν* is the kinematic viscosity. The calculated results demonstrate that the Rayleigh number for the melt of the alloy solidified on earth is about 10^3^ times larger than that for the melt of the alloy solidified in space, in other words, the Buoyancy-driven convective flow in the melt of the alloy solidified in space is much weaker than that in the melt of the alloy solidified on earth. It is in agreement with our previous research which demonstrates that the flow is laminar and the maximum flow velocity reaches a value about 6.7 μm/s when the alloy melt solidifies on earth, while it is only about 2.7 × 10^−3^ μm/s when the alloy melt solidifies in space.^36^ It also can be explained as: the density gradient in the melt caused by the gravity will diminish, even vanish under the microgravity environment in space, which leads to the significant decrease of the flow velocity.

**Fig. 5:**
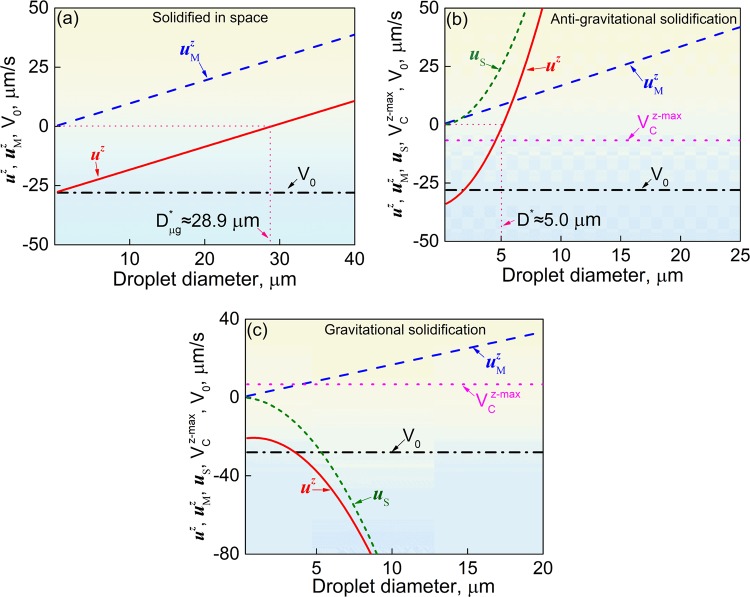
*Z*-vector for the resultant velocity (***u***^*z*^), the Marangoni (${\mathbf{u}}_{\mathrm{M}}^{\mathbf{z}}$), and Stokes (***u***_s_) motion velocity as a function of the diameter of the MP together with the maximum convective flow ($V_{\rm{C}}^{z - \max }$)^36^ and the withdraw velocity (**V**_0_) for the space sample **a**, anti-gravitational solidification sample **b**, and gravitational solidification sample **c**. D* is the critical diameter of the MP determined by ${\mathrm{|}}{\mathbf{u}}^{\mathbf{z}}(D^ \ast )| = 0$. The subscript μg represents the space sample

For the sample solidified in space, the Stokes motion of the MP and the Buoyancy-driven convective flow are both negligible, and the motion velocity of the MP relative to the solidification interface is approximately estimated by: ${\mathbf{u}}_{}^z \approx {\mathbf{u}}_{\rm{M}}^z + {\boldsymbol{V}}_0$. The calculations demonstrate that the MP with a diameter <28.9 μm can move toward the solidification interface (see Fig. 5a); this value is larger than the maximum diameter of the MP in the sample solidified in space (~28.0 μm, see Fig. 4). A steady state of solidification can be established and the sample solidified in space thus shows a well-dispersed microstructure.

For the anti-gravitational solidification sample on earth, the Stokes motion and Marangoni migration of the MP both cause the MP to move away from the solidification interface. The calculated result demonstrates that the MP with a diameter <5.0 μm can move toward the solid/liquid (S/L) interface (see Fig. 5b). The experimental results show that this value is much smaller than the maximum diameter of MP in the alloy sample solidified anti-gravitationally on earth, and a steady state of solidification cannot be established. The anti-gravitational solidification sample thus shows a microstructure with serious phase segregations.

For the gravitational solidification sample on earth, the Stokes sedimentation direction is opposite to the Marangoni migration direction of the MP. The Stokes motion of the MP thus is favorable for the establishment of the stable state of solidification and prevents the formation of phase-segregated microstructure. The calculated results demonstrate that, after the formation of the solidification interface, all the MP can move towards the solidification interface (see Fig. 5c), and a steady state of solidification can thus be established. The experimental results also indicate that the volume fraction and the average diameter of the MP along the axial direction of the sample almost keep constant except for the initial and final solidified ends (see Fig. 3). The inhomogeneous distribution of the MP in the initial and final solidified regions of the sample can be explained as: (1) Before the appearance of the solidification interface, some big MP may quickly migrate to the bottom of the sample due to the high Stokes motion velocity of the MP and the low cooling rate of the melt, which leads to the enrichment of MP in the bottom end region of the sample. (2) During the solidification of the top end region of the sample (the final solidified part of the sample), the Stokes motion of the MP causes them to move toward the solidification interface until they are captured by the solidification interface, while there is no other MP that migrate into this region, and a layer poor in MP thus formed.

**Fig. 6:**
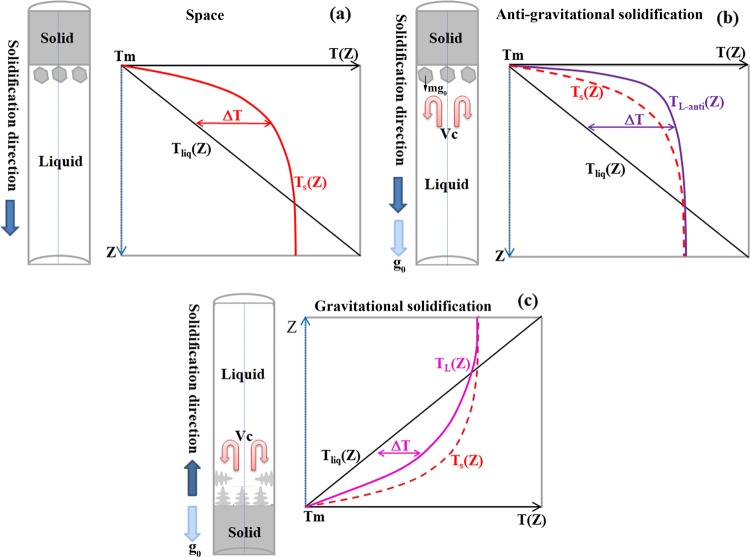
Sketch showing the variation of liquid undercooling ahead of the solid–liquid interface due to fluid flow for the space sample **a**, anti-gravitational solidification sample **b** and gravitational solidification sample **c**. mg_0_ is the gravity force of the α-Al crystal nuclei. *V*_C_ is the convective flow; *T*_m_ is the temperature of the solid–liquid interface; *T*_liq_(*Z*) is the temperature of the melt in front of the solidification interface; and *T*_S_(*Z*), *T*_L-anti_(*Z*), and *T*_L_(*Z*) are the liquidus temperatures of the melt in front of the solidification interface of the space sample, anti-gravitational solidification sample, and gravitational solidification sample, respectively. The solute concentration varies with the position in front of the solidification interface due to the fluid flow and the solute redistribution, and it will bring about the change of the liquidus temperature of the melt in front of the solidification interface

When the alloy solidifies in space, the absence of the gravity and the existence of the gap between the sample and the crucible favor the detachment between the alloy melt and the wall of the crucible and avoid the heterogeneous nucleation of the α-Al nuclei on the wall of the crucible, thus decreasing the defects on the surface of the sample and increasing the nucleation undercooling of α-Al nuclei. The increase of the nucleation undercooling leads to a higher nucleation rate of α-Al nuclei in the melt, promotes the equiaxed dendrite growth of the α-Al grains, and decreases the average grain size. Besides, with the growth of α-Al grains, solutes are rejected in front of the dendrite tip and a constitutional undercooling region may form (see Fig. 6a), and this is also propitious to the equiaxed dendrite growth of the α-Al grains. The microstructure of the sample solidified in space thus showing an equiaxed grain morphology, as shown in Fig. 1III(a). When the alloy solidifies on earth, the alloy melt contacts with the crucible wall, and the heterogeneous nucleation of α-Al may occur on the wall of the crucible. For the sample solidifying anti-gravitationally on earth, on the one hand, the melt convection flow and the gravity force may cause the α-Al crystal nuclei formed on the wall of the crucible fall off and move freely in the molten metal, thus promoting the multiplication of crystal nuclei; on the other hand, an upward fluid flow around the central region of the melt decrease the thickness of the solute boundary layer and increase the solute gradient in the liquid ahead of solidification interface, which leads to the enhancement of the constitutional undercooling degree^40^ (see Fig. 6b). The multiplication of crystal nuclei and the increase of constitutional undercooling degree both boost the formation of equiaxed structure, as shown in Fig. 1III(b). For the sample solidifying gravitationally on earth, the α-Al crystal nuclei first formed on the surface of the bottom region of the crucible and they are not easy to abscise from the crucible wall; those grains that grow parallel and opposite to the heat flow will advance rapidly, while other orientations tends to be overgrown due to mutual competition; it promotes the formation of the characteristic columnar zone, what is more, the upward fluid flow around the central region of the melt cause the increase of the extent of the solute boundary layer, which leads to a decrease of the solute gradient and the constitutional undercooling degree in front of the solidification interface (see Fig. 6c). Both of them promote the formation of columnar crystals, as shown in Fig. 1III(c).

**Fig. 7:**
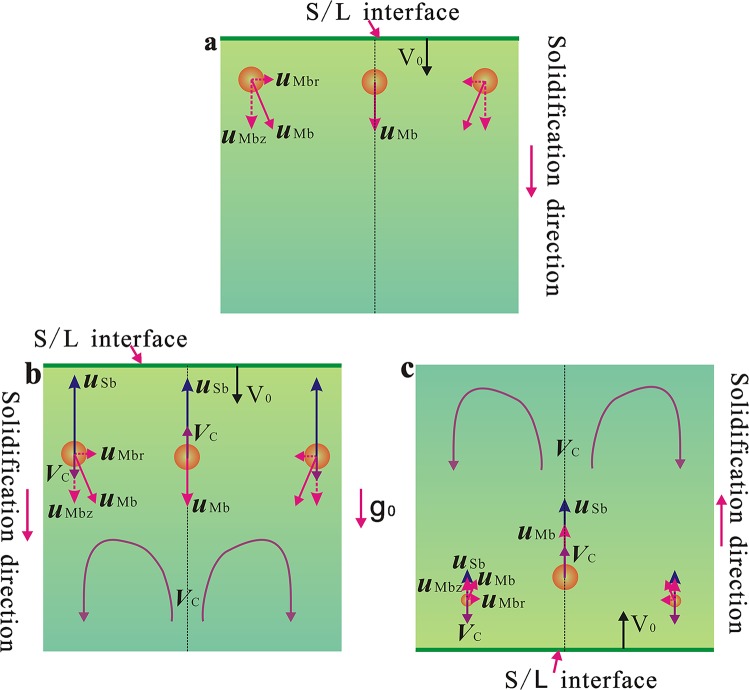
Schematics of the motions of the bubbles in the melt for the space sample **a**, anti-gravitational solidification sample **b**, and gravitational solidification sample **c**. ***u***_Sb_ and ***u***_Mb_ are the Stokes motion velocity and Marangoni moving velocity of the bubbles. ***u***_Mbz_ and ***u***_Mbr_ are the components of the Marangoni migration velocity of the bubbles along the axial direction and radial direction of the sample

During the solidification of aluminum alloys, the hydrogen is usually rejected from the solid phase into the liquid at the solid–liquid interface due to the enormous difference between the solubilities of hydrogen in the Al melt and in the α-Al. When the concentration of hydrogen in the liquid exceeds the supersaturation limit as dictated by Sievert’s law, the gas bubbles begin to nucleate.^41–43^ These bubbles then grow by the diffusion of solute in the matrix. They may also float due to the density difference between the matrix liquid and the bubbles under the action of gravity (Stokes motion) and migrate due to a temperature gradient (Marangoni motion). The motions of the bubbles have a dominating effect on the spatial distribution of the pores in the sample. For the sample solidified in space, the Stokes motion of the bubbles can be ignored and the bubbles move due to temperature gradient. If the axial direction component of the Marangoni motion velocity of the bubbles is larger than the advanced velocity of the solidification interface, the solidification interface cannot engulf the bubbles and the pores will not form in the sample, as shown in Fig. 7a and Fig. 1II(a). For the anti-gravitational solidification sample, the direction of the Stokes motion of the bubble is opposite to the advanced direction of the solidification interface along the axial direction. It boosts the migration of the bubble toward the solidification interface. In addition, the radial temperature gradient leads to the movement of the bubble toward the central axis of the sample. Thus the Stokes motion and the Marangoni motion promote the collision/coagulation between the bubbles and the formation of large gas cavities along the central axis of the sample, as shown in Fig. 7b and Fig. 1II(b). For the gravitational solidification sample, the Stokes motion and the Marangoni migration of the bubbles cause them to move away from the solidification interface, while the advancement of the solidification interface and the downward convection flow in the region close to the sample surface lead some bubbles to drift toward the S/L interface, which may result in the formation of many tiny pinholes in the sample (the size of the bubble is larger, its Stokes motion and Marangoni migration are quicker, so big size bubbles are not easy to be trapped by the S/L interface and only tiny pinholes exist in the sample), as shown in Fig. 7c and Fig. 1II(c).

In summary, directional solidification experiments were carried out with Al-Bi-Sn immiscible alloy in space and on earth. The solidification process and affecting mechanisms of microgravity on the microstructure evolution were discussed. The results indicate that the weak convective flow of the matrix melt and the diminished Stokes settlement of the MP under microgravity conditions promote the formation of a well-dispersed microstructure. The results also demonstrate that the microgravity conditions favor the detachment between the alloy melt and the wall of the crucible and prevent the heterogeneous nucleation of the α-Al nuclei on the wall of the crucible, thus decreasing the defects on the surface of the sample and increasing the nucleation undercooling of α-Al nuclei. The increase of the nucleation undercooling leads to the formation of equiaxed grain. Besides, the weak convective flow of the matrix melt and the insignificant Stokes motion of the bubbles under microgravity conditions reduce/diminish the porosity in the sample.

## Methods

### Sample preparation

**Fig. 8:**
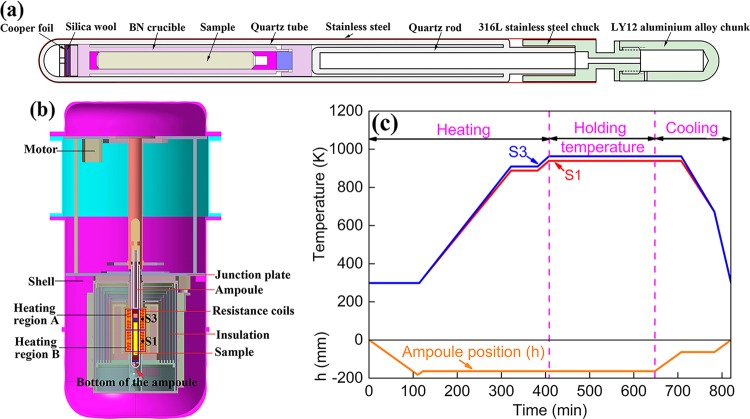
Schematic diagrams of the ampoule containing the Al-Bi-Sn sample **a** and the Material Experimental Furnace **b**. The designed experimental thermal control process **c**. S1 and S3 are the thermocouples monitoring the temperatures of the heating regions A and B, respectively. The ampoule position (h) in **c** corresponds to the distance between the bottom of the ampoule and the junction plate marked in **b**

The starting Al-Bi-Sn immiscible alloy was prepared by using pure Al (99.99%, mass percent, the same as below), Bi (99.99%), and Sn (99.99%) as the raw materials. The detailed procedures were as follows: the pure Al, Bi, and Sn were first melted and heated up to about 1073 K in a graphite crucible. Then the melt was held at this temperature and mixed for 30 min to form a single-phase liquid. After that, the melt was degassed and poured into a graphite mold to form cylindrical ingots of 12 mm in diameter and 150 mm in length. The composition of the ingots is Al–3.6wt.% Bi–1wt.% Sn, which is determined by using the inductively coupled plasma–atomic emission spectrometer. The ingots were machined into rods of $6.9_{ - 0.05}^0\,{\rm{mm}}$ in diameter and $57_{ - 0.05}^{0.05}\,{\rm{mm}}$ in length. Each of these rods was put into a boron nitride crucible of $75_{ - 0.5}^{0.5}\,{\rm{mm}}$ in depth, $7_0^{0.1}\,{\rm{mm}}$ in inner diameter, and $12_{ - 0.05}^{0.05}\,{\rm{mm}}$ in outer diameter. The gap between the ingot and the crucible can prevent the expanding crack of the boron nitride crucible due to the thermal expansion of the alloy sample during the heating and holding temperature process. The boron nitride crucible was then sealed into a quartz tube under a vacuum of about 1 × 10^−3^ Pa. Finally, the quartz tube was inserted into a steel tube of 0.1 mm in wall thickness to form an ampoule. The detailed structure of the ampoule is shown in Fig. 8a.

The directional solidification experiments were performed both in space and on earth by using the Material Experimental Furnace (see Fig. 8b). The temperatures of the heating regions A and B in the Material Experimental Furnace were monitored by the thermocouples S1 and S3. In order to reduce the Marangoni immigration of the MP, the temperature of the heating region A was set 15 °C higher than that for the heating region B during the holding temperature period and the cooling period. The detailed experimental procedures are shown in Fig. 8c. The microgravity solidification experiment was conducted onboard the Tiangong 2 space laboratory of China during its flight in October 2016. The microgravity level was about 10^−3^ *g*_0_ with *g*_0_ being the gravitational acceleration on earth. Two ground samples were solidified by following exactly the same procedures: one sample ampoule was withdrawn against the gravity direction (called “anti-gravitational solidification”) and the other one was withdrawn along the gravity direction (called “gravitational solidification”). The average cooling rates from the binodal line temperature (658.9 °C) to the S/L interface temperature (643.5 °C) are ~0.026 and ~0.029 °C/s for the alloy solidified in space and on ground, respectively.^36^

### Sample characterizations

After solidification, the samples were sectioned longitudinally and the metallographic specimens were prepared. The optical microscope, SEM equipped with EDS, X-ray tomography, and the SISC IAS V 8.0 software developed by the Chinese Academy of Sciences were employed to analyze the macro-/micro-structures of these samples, e.g., the appearance of the sample surface, the distribution of the gas pore, the grain morphology, the size distribution and spatial distribution of the MP particles, etc.

## Supplementary information

nr-reporting-summary-NPJMGRAV-00389

## Data Availability

The datasets generated and analyzed during the current study are available from the corresponding author on reasonable request.
